# Recurrent common bile duct stones as a late complication of endoscopic sphincterotomy

**DOI:** 10.1186/s12876-018-0765-3

**Published:** 2018-03-15

**Authors:** Tatenda C. Nzenza, Yahya Al-Habbal, Glen R. Guerra, S. Manolas, Tuck Yong, Trevor McQuillan

**Affiliations:** Department of Hepatobiliary Surgery, Northern Health, Epping, VIC 3076 Australia

**Keywords:** ERCP, Endoscopic sphincterotomy, Recurrent bile duct stones

## Abstract

**Background:**

Endoscopic retrograde cholangiopancreatography (ERCP) with endoscopic sphincterotomy (ES) has become well established as a modality for the management of common bile duct stones (CBDS), especially in the setting of associated cholangitis. Our study aims to determine the rate of long term morbidity of recurrent CBDS post ES.

**Methods:**

A retrospective analysis of patients who underwent ERCP and ES (ERCP+ES) was undertaken on a prospectively maintained database from 1998 to 2012 at the Northern Hospital, Melbourne. Primary CBDS were defined as those detected at least 6 months after complete clearance of the CBD. Prior cholecystectomy was a requirement for inclusion and patients with primary CBD stones in the setting of an intact sphincter were excluded.

**Results:**

A total of 1148 patients underwent ERCP, of which 573 had an ES. Fifty-one patients underwent an ES prior to developing primary CBDS (8.9%). The time to recurrence ranged from 6 months to 15 years (mean 3.3 years). The number of procedures per patient ranged from 2 to 11, with 51% requiring 3 or more ERCPs. Factors associated with primary CBDS recurrence included a dilated CBD > 12 mm, stricture of the major papilla post ES to 2 - 5 mm and presence of the ampulla within or on the edge of a duodenal diverticulum.

**Conclusion:**

The results demonstrate that ERCP + ES has an inherent long-term complication of recurrent primary CBDS formation. While this can be managed with repeat ERCP, the advent of laparoscopic bile duct exploration should lead us to re-examine the role of ERCP + ES in younger patients.

## Background

Endoscopic retrograde cholangiopancreatography (ERCP) with endoscopic sphincterotomy (ES) was introduced in 1974, initially aimed at frail and elderly patients, deemed unfit for surgery [1]. However, it has become well established as the first line therapeutic modality in the management of CBDS across many centres.

Various studies have described complications associated with ES, both in the short and long term. Well documented early complications of ES include pancreatitis, bleeding, and cholangitis [2–4]. Long term complications include papillary stenosis or recurrent CBDS, with the latter being a burden to both the patient and healthcare industry.

The aim of this study was to determine the rate of recurrent common bile duct stones post ERCP and ES.

## Method

A retrospective analysis of patients who underwent ERCP and ES from 1998 to 2012 at the Northern Hospital, Melbourne, Australia, was undertaken on a prospectively maintained database. The study was approved by the Northern Health Human Research Ethics Committee (AU/14/F1D818) and supported by a Northern Health small research grant.

All patients 18 years old or over, treated between 1 January 1998 and 31 December 2012, having undergone an ERCP with or without ES, were included. Cholecystectomy performed previously or within the first 2 months following ES was a requirement for inclusion.

Recurrent primary CBDS were defined as those detected more than 6 months following initial ERCP with complete duct clearance. Patients who presented with primary CBDS at least 6 months’ post cholecystectomy with an intact sphincter were excluded from the analysis.

The database was interrogated to obtain data for this study, with further information gleaned from archival medical records, correspondence from local practitioners, and via reports from other hospitals outside of the Northern Health network. Collected data focused on patient demographics, symptoms on presentation and ERCP findings. This included the size of the CBD, presence of ampullary stenosis, and classification of a duodenal diverticulum where present (duodenal diverticulum is classified as Type “A” - Ampulla on edge or within diverticulum; and Type “B” Ampulla distant to diverticulum), given the different stone recurrence rates associated with each type [5]. Episodes of recurrent primary CBDS were recorded, with the time to recurrence calculated from the index ERCP to the subsequent ERCP. Statistical analysis was undertaken with Graph-pad prism® v6, with the Chi-squared test utilised to assess significance, set at *p* < 0.05.

## Results

A total of 1148 consecutive patients underwent an ERCP during the study period (Table 1). Of these, 573 (49.9%) had an ES. There were 724 female patients (63%) and 424 male patients (37%) with a female-to-male ration of 1.7:1. The median patient age was 67 years at the time of the index ERCP (range 40–105 years).

**Table 1:** Demographics of the study cohort

	ERCP	ERCP + ES	Recurrent CBDS
Female	724	342	33
Male	424	231	18
Total	1148	573	51

**Fig. 1:**
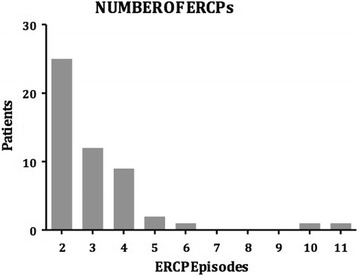
Illustrating the number of patients that underwent multiple ERCP episodes

**Fig. 2:**
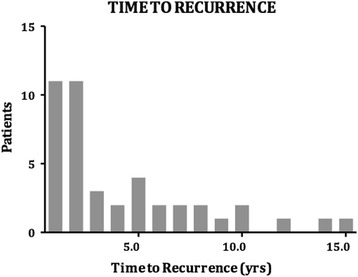
Plot showing the time to recurrence for patients during the follow up period

**Fig. 3:**
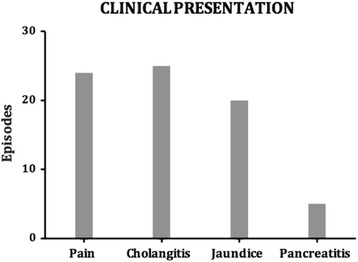
Range of clinical presentations for the various episode in our series

**Fig. 4:**
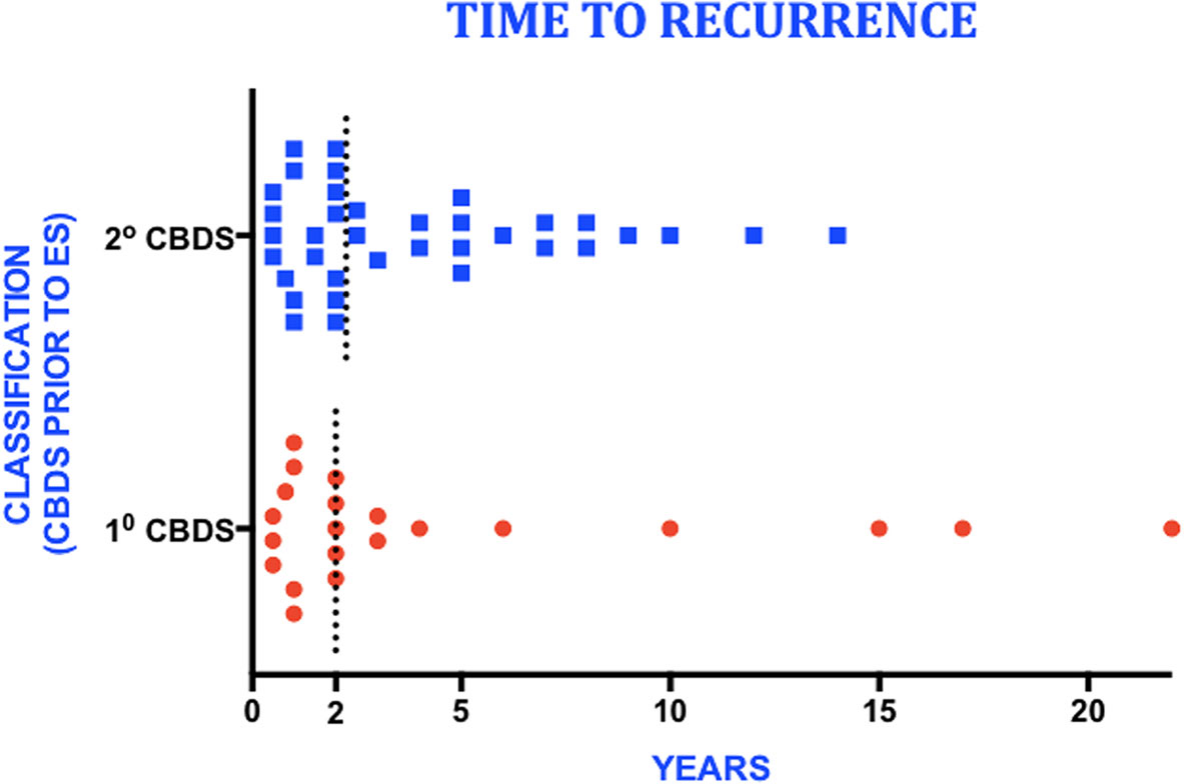
Plot showing the time to recurrence for primary and secondary bile duct stones

Sixty patients presented with recurrent primary CBDS, of which 51 (8.9%) occurred at least 6 months following ES and cholecystectomy. The other 9 patients developed primary CBDS 6 months following cholecystectomy in the setting of an intact sphincter and were excluded from the analysis. There was a total of 68 episodes of primary CBDS, which were addressed with 162 ERCPs. Twenty-six patients required 3 or more procedures (51% of patients with stone recurrence) (Fig. 1). The maximum number of ERCP procedures in a single patient was 11. The time to recurrence ranged from 6 months to 15 years (mean 3.3 years) (Figs. 2 and 3). The clinical presentation heralding primary CBDS recurrence was varied and included pain, cholangitis, jaundice, pancreatitis or a combination of the above (Fig. 4). Pain and cholangitis were the two most common presentation.

### Risk factors

The presence of a peri-ampullary diverticulum was associated with a higher rate of CBDS recurrence. This rate was dependent on the classification of the diverticulum as either type A where the major papilla is located on the inner rim or inside the diverticulum or type B where the major papilla is distant to the diverticulum. Type A and type B diverticula were associated with a CBDS recurrence rate of 25% (RR 2.3, *p* = 0.07) and 7% (RR 1.9, *p* = 0.27) respectively (Table 2). Consequently, the presence of a type A diverticulum was associated with a statistically significant higher rate of primary CBDS recurrence, with a RR of 2.3.

**Table 2:** The association of a peri-ampullary diverticulum with recurrent primary CBDS

	ERCP + ES	Recurrent Primary CBDS	RR
Diverticulum	16%	32%	RR 2.2 (*p = 0.002*)
Type A	12%	25%	RR 2.3 (*p = 0.007*)
Type B	4%	7%	RR 1.9 (*p = 0.27*)

Another endoscopic finding of significance was that of papillary stenosis post ES. This was noted in 11 patients (2%), of which 5 (46%) developed recurrent primary CBDS. CBD diameter was also noted to be associated with primary CBDS recurrence. A CBD diameter <  12 mm had a recurrence rate of 20% (RR 0.50; *p* = 0.053) compared with 34% for patients with a CBD of 12–15 mm (RR 1.19; *p* = 0.54), and 46% for those with a CBD diameter >  15 mm (RR 1.7; *p* = 0.039). Consequently, a dilated CBD showed an incrementally increased risk of recurrent primary CBDS (Table 3).

**Table 3:** The association of CBD diameter with recurrent primary CBDS

	ERCP + ES	Recurrent Primary CBDs	RR
< 12 mm	50%	20%	RR 0.50 (*p* = 0.053)
12–15 mm	27%	34%	RR 1.19 (*p* = 0.54)
> 15 mm	23%	46%	RR 1.7 (*p* = 0.039)

## Discussion

Cholelithiasis and cholecystitis are among the most common surgical conditions, with approximately 19,000 cases of cholecystectomy performed each year in Australia. Ten percent of patients with cholelithiasis will have associated CBD stones, and while management of gallstones and cholecystitis have clear guidelines, the management of CBD stones has historically been more controversial.

The management options of CBDS without cholangitis can be operative, endoscopic or radiological. Operative approaches include open (trans-cystic or choledochotomy), laparoscopic (trans-cystic or choledochotomy) or endoscopic (ERCP), which was first described in 1974 [1]. Percutaneous trans hepatic decompression, is a radiological approach that is utilized less frequently and carries significant risk.

In a Cochrane review from 2013, 16 randomised clinical trials with a total of 1758 patients were examined, comparing laparoscopic and open CBD exploration with ERCP in the management of CBD stones. There was no significant difference between these approaches, in both morbidity and mortality. However, open CBD exploration was associated with a higher clearance rate (94% vs 84%) [2].

In the setting of cholangitis, the management approach is mainly ERCP [2, 6]. In a single centre study by Poh et al., it was found that ERCP was more efficient in clearing the bile duct. This paper reported a total of 182 patients that had an emergency laparoscopic cholecystectomy, and received intervention for choledocholithiasis. The duct clearance rate was found to be 63% in the laparoscopic bile duct exploration group, compared to 86% in the ERCP group [7]. However, stone clearance rates by laparoscopic and open techniques have been reported to be as high as 98% in other papers. In a retrospective cohort study by Lee et al., data from a single centre was collected between 1997 and 2011. Laparoscopic bile duct exploration was attempted in 157 patients with conversion to open in 5 (3.2%). Bile duct exploration was done through a choledochotomy with a clearance rate of 98% [8]. The wide variation between studies, can stem from differences in local experience and resource availability. Despite this, ERCP is still the most commonly used modality of treatment for CBD stones in Australia, as reported in a recent narrative review by March et al. [9].

There are several studies that report on complications of ERCP, especially in association with a sphincterotomy [4, 10, 11]. These include pancreatitis, bleeding, perforation and cholangitis in the short term and recurrent CBDS in the long term. Physiologically, the predominant theory behind the development of recurrent primary CBDS post ES, is the reflux of duodenal contents into the biliary system leading to bacterial colonization and subsequent stone formation [12]. Additionally, though there is no high level evidence of ES causing atypia or malignancy, there is some evidence it causes reactive and proliferative changes in the CBD [13].

As an alternative to sphincterotomy, the ampulla can be dilated endoscopically. A recent meta-analysis by Jin et al. demonstrated no short term difference in outcome when comparing endoscopic sphincterotomy to balloon dilatation. Both techniques showed equal rates of stone retrieval, with no statistically significant difference in the incidence of overall adverse events (7.9% vs 10.7%, *P* = 0.25), post-ERCP pancreatitis (4.0% vs 5.0%, *P* = 0.54), haemorrhage (1.7% vs 2.8%, *P* = 0.32), perforation (0.3% vs 0.9%, *P* = 0.35) or acute cholangitis (1.3% vs 1.3%, *P* = 0.92). Long-term complications were not reported in that meta-analysis [14], however, Tsujino et al. reported that balloon dilatation alone was associated with recurrent stone disease in 13.5% over a 10-year follow up period [15].

In general, the recurrence rate of CBDS quoted in the literature ranges between 4 and 25% [16, 17]. This wide discrepancy likely reflects selection bias and differences in follow up periods. Kim et al. retrospectively reviewed 101 patients who underwent an ERCP for CBDS with a mean follow up period of 25 months. The CBDS recurrence rate was found to be 5.8–6.9%. There was no statistical difference between stone recurrence associated with ES and balloon dilatation, and time to recurrence ranged from 10 to 42 months. Univariate analysis demonstrated that the number of ERCP sessions to clear CBD stones, the angle between proximal and distal CBD of < 135 degrees, use of mechanical lithotripsy, and the presence of a peri- ampullary diverticulum were statistically significant risk factors. Multivariate analysis revealed that the presence of a peri-ampullary diverticulum was the only independent risk factor for CBD stone recurrence [18]. In a further study by Costamagna et al., 529 patients underwent an ERCP + ES with a minimum 5 year follow up. Stone recurrence was reported as 11.1%, with a statistically significant association with a dilated CBD duct > 22 mm [4].

Our study has demonstrated that ERCP + ES has an inherent long term complication rate of recurrent primary CBDS formation. The rate of 8.9% is in keeping with those reported in the literature. A significantly increased risk is associated with the presence of a type A peri-ampullary diverticulum and a dilated common bile duct of > 15 mm, also in keeping with the literature. Sphincterotomy or dilatation of the ampulla is generally undertaken at the time of ERCP to facilitate CBDS extraction. This can damage the physiological barrier provided by the sphincter of Oddi, allowing reflux of enteric contents and bacteria into the CBD, predisposing to the formation of recurrent primary CBD stones [19, 20].

An alternative minimally invasive method to manage CBDS and avoid the long term complication of ES, is to extract CBDS laparoscopically either trans-cystically or via a choledochotomy. This operative approach has its own complications, with choledochotomy reported to have an increased risk of bleeding, CBD stricturing and bile leak [21, 22]. Hong et al compared laparoscopic cholecystectomy and ERCP (LC+ERCP) to laparoscopic cholecystectomy and operative CBD exploration (LC+BDE). They randomised 234 patients with no statistically significant difference in outcome, including operating time, success rate, postoperative complications, retained stone rate, length of stay or hospital cost [23]. However, all bile duct explorations were undertaken via a choledochotomy. Use of choledochoscopy or imaging guided baskets via the transcystic approach may lead to different results.

There were several limitations to our study. Firstly, it is retrospective, carrying an inherent bias in data collection, and being limited by the quality of the available clinical notes. Information concerning late complications would have ideally been obtained from multiple sources to ensure complete and accurate data collection. Secondly, there are several hospital-based ERCP services in close proximity. Consequently, there is a possibility of under representing the true rate of recurrent primary CBDS, as patients may have presented to other surrounding centres. Lastly, the study group consisted of a relatively elderly cohort, which may have also impacted on the rate of recurrent primary CBDS.

## Conclusion

ERCP + ES currently remains the most commonly performed procedure for CBDS management. This is associated with a high recurrence rate of CBDS. Other emerging treatment modalities such as laparoscopic trans-cystic bile duct exploration (LTCBDE) and laparoscopic bile duct exploration (LBDE) may be associated with lower rates of recurrent CBD stones. A well structured study comparing the long term outcomes of LTCBDE or LBDE to those of ES would assist in clarifying the best option for the management of CBDS.

## Abbreviations

CBD: Common bile duct
CBDS: Common bile duct stone
ERCP: Endoscopic retrograde pancreaticoduodenography
ES: Endoscopic sphincterotomy

## References

[1] Kawai K (1974). Endoscopic sphincterotomy of the ampulla of Vater. Gastrointest Endosc.

[2] Dasari BV, et al. Surgical versus endoscopic treatment of bile duct stones. Cochrane Database Syst Rev. 2013;(9): p. CD003327. 10.1002/14651858.CD003327.pub3 23999986

[3] Enochsson L (2004). Intraoperative endoscopic retrograde cholangiopancreatography (ERCP) to remove common bile duct stones during routine laparoscopic cholecystectomy does not prolong hospitalization: a 2-year experience. Surg Endosc.

[4] Costamagna G (2002). Long-term follow-up of patients after endoscopic sphincterotomy for choledocholithiasis, and risk factors for recurrence. Endoscopy.

[5] Kim CW (2013). Size and type of periampullary duodenal diverticula are associated with bile duct diameter and recurrence of bile duct stones. J Gastroenterol Hepatol.

[6] Abu Dayyeh BK (2012). Endoscopic sphincterotomy: indications, techniques, and adverse events. Tech gas int end.

[7] Poh B (2014). Management of choledocholithiasis in an emergency cohort undergoing laparoscopic cholecystectomy: a single-centre experience. HPB (Oxford).

[8] Lee HM, Min SK, Lee HK (2014). Long-term results of laparoscopic common bile duct exploration by choledochotomy for choledocholithiasis: 15-year experience from a single center. Ann Surg Treat Res.

[9] March B, Burnett D, Gani J (2016). Single-stage laparoscopic cholecystectomy and intraoperative endoscopic retrograde cholangiopancreatography: is this strategy feasible in Australia?. ANZ J Surg.

[10] Bergman JJ (1996). Long-term follow-up after endoscopic sphincterotomy for bile duct stones in patients younger than 60 years of age. Gastrointest Endosc.

[11] Tham TC, Carr-Locke DL, Collins JS (1997). Endoscopic sphincterotomy in the young patient: is there cause for concern?. Gut.

[12] Tsujino T (2007). Endoscopic papillary balloon dilation for bile duct stone: immediate and long-term outcomes in 1000 patients. Clin Gastroenterol Hepatol.

[13] Kalaitzis J (2012). Effects of endoscopic sphincterotomy on biliary epithelium: a case-control study. World J Gastroenterol.

[14] Jin PP (2014). Endoscopic papillary large balloon dilation vs endoscopic sphincterotomy for retrieval of common bile duct stones: a meta-analysis. World J Gastroenterol.

[15] Tsujino T (2008). Long-Term Outcomes (Mean Follow-Up Period > 10 Years) of Endoscopic Papillary Balloon Dilation for Bile Duct Stones. Gastrointest Endosc.

[16] Prat F (1996). Biliary symptoms and complications more than 8 years after endoscopic sphincterotomy for choledocholithiasis. Gastroenterology.

[17] Freeman ML (1996). Complications of endoscopic biliary sphincterotomy. N Engl J Med.

[18] Kim KY (2013). Late complications and stone recurrence rates after bile duct stone removal by endoscopic Sphincterotomy and large balloon dilation are similar to those after endoscopic sphincterotomy alone. Clin Endosc.

[19] Toouli J (2009). Sphincter of Oddi: function, dysfunction, and its management. J Gastroenterol Hepatol.

[20] Mandryka Y (2006). Bile duct infections as a late complication after endoscopic sphincterotomy. Pol Merkur Lekarski.

[21] Wills VL (2002). Complications of biliary T-tubes after choledochotomy. ANZ J Surg.

[22] Leida Z (2008). A randomized comparison of primary closure and T-tube drainage of the common bile duct after laparoscopic choledochotomy. Surg Endosc.

[23] Hong DF, Xin Y, Chen DW (2006). Comparison of laparoscopic cholecystectomy combined with intraoperative endoscopic sphincterotomy and laparoscopic exploration of the common bile duct for cholecystocholedocholithiasis. Surg Endosc.

